# Robust half-metallicity and topological aspects in two-dimensional Cu-TPyB

**DOI:** 10.1038/srep14098

**Published:** 2015-09-14

**Authors:** Xiaoming Zhang, Mingwen Zhao

**Affiliations:** 1 School of Physics and State Key Laboratory of Crystal Materials, Shandong University, Jinan 250100, Shandong, China

## Abstract

Half-metallicity due to the coexistence of metallic nature for one spin component and insulating nature for the other is a base of spintronics devices, but was only achieved in few materials. From first-principles calculations, we demonstrate that a recently-synthesized two-dimensional organometallic framework of 1,3,5-tris(pyridyl)benzene and Cu atoms (Cu-TPyB) has robust half-metallicity. High electron velocity in one spin channel at Dirac point and a relatively large band gap in the other make the material meeting the demand of filtering the current into a single spin component. Moreover, spin-orbit coupling induces topologically nontrivial band gaps in the vicinity of the Fermi level, which are implementable for achieving quantum anomalous Hall effect in a low temperature range (<8 K).

Spintronics is a multidisciplinary field that involves the study of active control and manipulation of spin degrees of freedom in solid-state systems. A stimulating innovation in this area is the generation of 100% spin-polarized current. Half-metallic ferromagnets, which have a metallic nature for one spin component but are insulating for the other, can filter the charge current into a single spin channel and act as spin injectors^1^,^2^. Additionally, topological quantum spintronics, with the advantage of low-power-consumption, has recently aroused extensive interests of researchers. Magnetic topological insulators (TI), which have quantum anomalous Hall (QAH) states with the internal magnetization breaking time-reversal symmetry and spin-orbit coupling (SOC) opening band gaps, can give rise to quantized Hall conductivities and meet the demands of topological quantum spintronics^3^. Hence, searching for the materials with robust half-metallicity and TI states is an important goal for developing (topological quantum) spintronics devices.

Up to now, tremendous efforts have been devoted to searching for half-metals. The proposed candidates range from transition-metal(TM)-containing materials, such as organometallic sandwich molecular wires^4^,^5^, TM-polyporphyrin^6^,^7^, and TM-doped semiconductors^8^,^9^,^10^, to metal-free compounds, such as graphene based system^11^, DTPA porous sheet^12^,^13^ and graphitic carbon nitrides^14^,^15^. The direct observations of half-metallicity in experiments were achieved by spin-resolved photoemission measurements^16^,^17^. Magnetic TIs have also been predicted in inorganic^3^,^18^, organic^19^,^20^, and TM-free compouds^21^. In very recent experiments, QAH effect was achieved successfully in a Cr-doped (Bi,Sb)_2_Te_3_ topological insulator, but the operating temperature is quite low (~30 mK)^3^. Increasing the operating temperature is the key for practical applications.

Recently, a family of two-dimensional (2D) hexagonal frameworks have been synthesized, such as nickel bis(dithiolene) nanosheet^22^, (Ni_3_(HITP)_2_)^23^, and cobalt or platinum dithiolene complexes^24^,^25^. The topologically nontrivial states which are mainly contributed by the fourfold-coordinated TM atoms have been predicted in these materials^26^,^27^,^28^, and some of them may be Chern insulators, which are implementable for achieving QAH effect. The growth of another stable 2D organometallic framework consisting of 1,3,5-tris(pyridyl)benzene and Cu atoms, Cu-TPyB, with twofold Cu-pyridyl coordination (Fig. 1) on Au(111) metal surfaces was reported in recent experiments^29^. The remarkable thermal stability of the hexagonal Cu-TPyB lattice is quite promising for electronic devices applications.

In this contribution, we theoretically demonstrate that the interaction between Cu and TPyB ligands leads to a stable ferromagnetic (FM) spin-polarization and robust half-metallicity of the Cu-TPyB framework, although neither isolated Cu atom nor the TPyB ligand is spin-polarized. The high electron velocity in one spin channel at Dirac point and a relatively large band gap in the other meet the demand of filtering the current into a single spin component. In contrast to most of the organometallic frameworks reported in previous works, the exotic properties of the Cu-TPyB are related to the unique π-orbits of the TPyB ligands, which lead to two Dirac bands sandwiched by two topological flat bands in the vicinity of the Fermi level. More interestingly, moderate SOC opens topologically nontrivial band gaps between the upper flat band and the upper Dirac band and between the two Dirac bands, which may be used for hosting QAH effects at temperatures much higher than that has been achieved in Cr-doped (Bi,Sb)_2_Te_3_. Both the half-metallicity and topological nontriviality are robust against external strain. These features make the Cu-TPyB an ideal material for the applications in future (topological quantum) spintronics devices.

## Results

**Figure 1:**
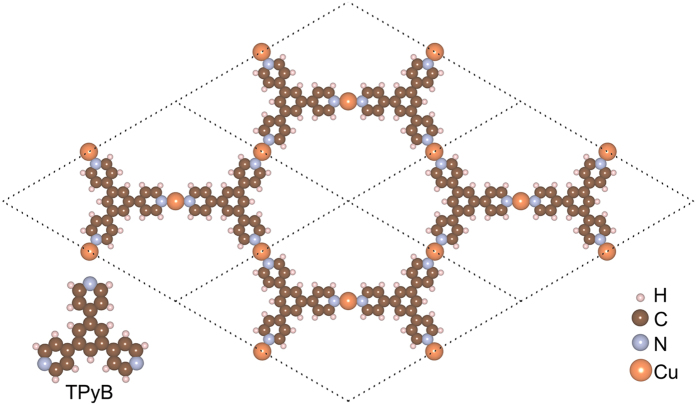
Schematic representation of Cu-TPyB with twofold Cu-pyridyl coordinations. The organic ligand of TPyB (1,3,5-tris(pyridyl)benzene) is presented in the inset.

The 2D organometallic framework was assembled by 1,3,5-tris(pyridyl)benzene (TPyB) and Cu, forming a twofold Cu-pyridyl coordination with a chemical formula of C_42_N_6_H_30_Cu_3_ and 261 valence electrons in one unit cell (referred to as Cu-TPyB), as shown in Fig. 1. After structural relaxation, the planar configuration and six-fold rotational symmetry are both preserved, displaying features of a structurally perfect hexagonal network. The N-Cu distance is about 1.85 Å, suggesting the chemical binding between Cu and pyridyls. The optimized lattice constant of freestanding Cu-TPyB, *a*_*0 *_= 26.39 Å, is slightly shorter than the experimental value (27.3 ~ 27.6 Å), which can be attributed to the lack of interactions between Cu-TPyB and Au(111) substrate in our calculations.

### Magnetic configurations

**Figure 2:**
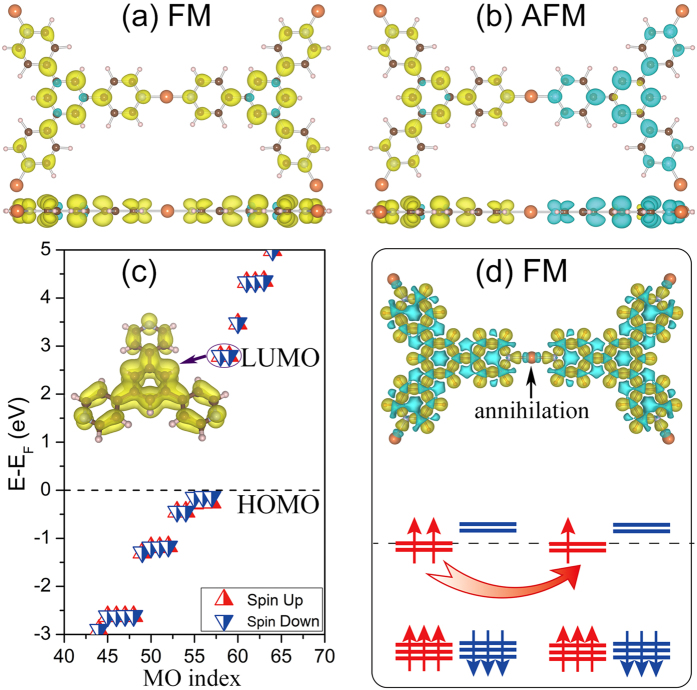
Magnetic properties of Cu-TPyB. (**a,b**) The spatial distributions of spin-polarized electron density in (**a**) FM and (**b**) AFM states. Different colors represent different electron spins. (**c**) Energy spectrum of the MOs of an isolated TPyB ligand in the vicinity of the Fermi level. Only the valence electrons are counted in the MO index. The energy at the Fermi level was set to zero. The partial charge density isosurfaces of the LUMOs are shown in the inset. (**d**) The exchange mechanism of spin-resolved energy levels between TPyB ligands, virtual hopping is allowed for the FM arrangement. Spatial distribution of the differential charge density with respect to individual atoms is shown in the inset of (**d**). The annihilation and creation of electrons are represented by blue and yellow, respectively. The electron density isosurface values are all set to 0.002 Å^−3^.

The odd number of electrons in one unit cell reminds us if the Cu-TPyB has a spin-polarized ground state. Starting from different spin configurations, self-consistent calculations give two types of magnetic orderings, as indicated by the spatial distributions of spin-polarized electronic density shown in Fig. 2a,b. One has the local magnetic moments aligned in a parallel way, while the local magnetic moments in the other align in an anti-parallel way, which correspond to FM and antiferromagnetic (AFM) orderings, respectively. The spin-polarization comes mainly from the *p*_*z*_ orbits of carbon and nitrogen atoms in the TPyB ligands, resulting in a magnetic moment of 3.0 μ_B_ for FM and 0.0 μ_B_ for AFM in one unit cell. Total energy comparison indicates that the FM ordering is more stable than the AFM and nonmagnetic (NM) states by about 59.34 and 121.23 meV/unit cell, respectively. Although the energy differences normalized to atom are much lower than the room temperature, the local magnetic moments in the TPyB unit are robust against structural distortion. The thermal stability of the FM ordering of these local magnetic moments can be evaluated using Monte Carlo simulations within the *Ising* model, which give a Curie temperature of about 137 ~ 151 k. More details can be seen in the Supplementary Information (SI) online.

The energetic stability of FM ordering is understandable from the molecular orbital (MO) analysis of the TPyB ligand^30^. The energy eigenvalues of the MOs in the vicinity of the Fermi level of an isolated TPyB ligand calculated from DFT are plotted in Fig. 2c. The highest occupied molecular orbit (HOMO) is threefold-degenerate with the two spin branches occupied equally, while the lowest unoccupied molecular orbit (LUMO) is doubly-degenerate, both of which stem from the *p*_*z*_-orbits of carbon and nitrogen atoms, as shown in the inset of Fig. 2c. When they are jointed together via N-Cu-N bridges, electrons transfer from Cu to the TPyB ligands, as indicated by the differential electron density distribution of the Cu-TPyB lattice (see the inset of Fig. 2d). Cu atom in a linear coordination is typically in the Cu^+^ state. According to Pauli exclusion principle, three electrons of the three Cu atoms in one Cu-TPyB unit cell occupy the doubly-degenerate LUMOs of the two TPyB ligands with the same spin direction (see Fig. 2d), leading to a net magnetic moment of 3.0 μ_B_. The partially-filled spin-up states will act as acceptor levels mutually, allowing a virtual hopping for FM arrangement, which gives rise to a stable FM configuration^13^,^31^.

### Half-metallicity

**Figure 3:**
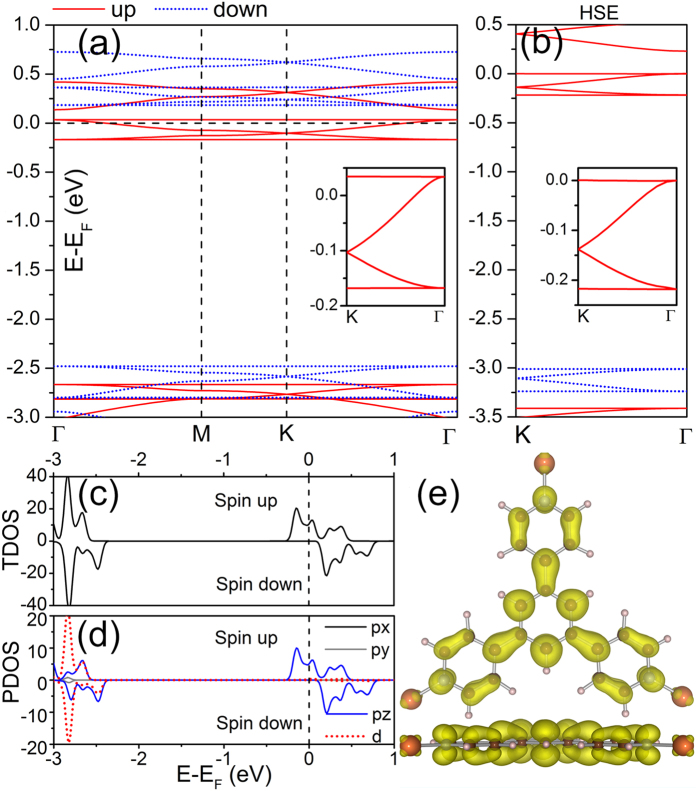
Electronic properties of Cu-TPyB. (**a,b**) Spin-polarized band structures obtained from (**a**) GGA-PBE and (**b**) HSE calculations. Enlarged views of the four bands near the Fermi level are presented in the insets. (**c**) Total and (**d**) orbital-resolved electronic density of states. (**e**) The partial charge density isosurfaces decomposed to the two flat bands and the two Dirac bands in the energy range from *E*_*F*_ - 0.2 to *E*_*F *_ + 0.1 eV with the isosurface value of 0.002 Å^−3^.

The electronic band structures of the ferromagnetic Cu-TPyB are then calculated. The characteristics of half-metallicity are quite obvious from the spin-resolved band lines along the highly-symmetric points in BZ, as shown in Fig. 3a. The electrons in the spin-up channel have metallic nature while the spin-down channel is insulating with a trivial band gap of 2.66 eV. Interestingly, the bands near the Fermi level are composed of two Dirac bands sandwiched by two flat bands. Without SOC, the flat band and Dirac band are degenerate at Γ points, while the two Dirac bands are degenerate at K points with linear dispersion (Dirac cone). The Fermi velocity (*v*) of electrons at the Dirac cone calculated from the expression 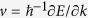 along the Γ-K direction is about 0.9 × 10^5^ *m/s*, which is of the same order as the electron saturation velocity in a silicene (~10^5 ^*m/s*)^32^. This suggests that the carriers in this Dirac bands may have high mobility as in silicene when the Fermi level is shifted to the Dirac point, e.g. by applying a strong electric field^33^,^34^,^35^. The high group velocity and a large band gap in different spin channels meet the demand of filtering the current into a single spin channel and thus are quite promising for spintronics devices applications.

The abundant states for spin-up and a large band gap for spin-down channel near the Fermi level can be seen more clearly from the spin-resolved total electronic density of states (TDOS) shown in Fig. 3c. The electron density of states projected onto different atomic orbits (PDOS) show that the half-metallicity comes mainly from the non-local *p*_*z*_ atomic orbits and minor from the *d* atomic orbit (see Fig. 3d), which indicates that the conducting bands have conjugated π-bonding characteristics, coinciding with the high electron mobility in the Cu-TPyB framework. This feature is further confirmed by the partial charge density decomposed to the four bands in the vicinity of the Fermi level, as shown in Fig. 3e. The profile and location of the isosurfaces exhibit the features of the *p*_*z*_-orbits of carbon and nitrogen atoms, which are consistent with the LUMOs of the TPyB ligands.

To further confirm the half-metallicity, we also employed a hybrid Heyd-Scuseria-Ernzerhof (HSE) functional^36^,^37^,^38^, which can reproduce well the electronic band gap of semiconducting materials. It is found that the band structures of the Cu-TPyB resemble those obtained from GGA-PBE strategy very much in the half-metallicity nature, except the larger splitting between the two spin channels, as shown in Fig. 3b. The band gap of the spin-down channel is enlarged to about 3.67 eV, which facilitates the stability of the half-metallicity. The FM state is energetically more favorable than the AFM state by about 40.81 meV/unit cell. It is noteworthy at 0 K the Fermi level of the Cu-TPyB lattice will right locate at the tip of the upper Dirac band, in resonance with the flat band, where the electron velocity is vanishing. The half-metallicity of the Cu-TPyB lattice will be quenched, even though the FM ordering remains robust. However, with the ability of realizing electrons and holes doping in experiment, gate voltage^33^ can move the Fermi level to the region near the Dirac cone and turn on the half-metallicity, which makes the Cu-TPyB an ideal spin-current control switch.

### Topological nontriviality

**Figure 4:**
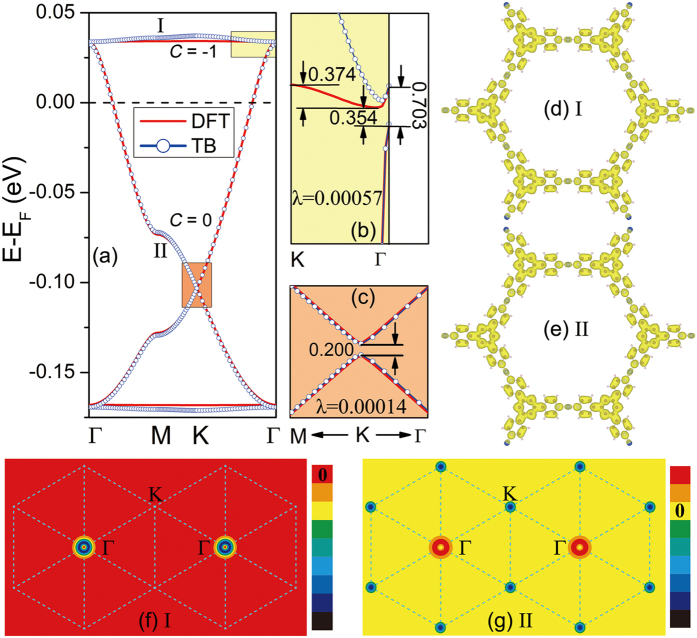
Topological properties of Cu-TPyB. (**a**) A comparison between DFT and TB calculations for the four bands calculated with SOC. Enlarged views of the colored areas in (**a**) are presented in (**b**) and (**c**) with the unit of band gaps in meV. The 2D distribution of (**d,e**) band decomposed charge density in real space and (**f,g**) Berry curvature in momentum space for the bands I and II labeled in (**a**).

Topological insulators with stable ferromagnetism are implementable for achieving QAH effect which occurs without need of external magnetic fields. We take the SOC into account to reveal the topological aspects of the electronic structures in the Cu-TPyB. As a benchmark, we first calculated the SOC gap in graphene and found that it is only 0.008 meV, close to the value of previous works^39^. Using the same method, we calculated the band structures of Cu-TPyB in the vicinity of the Fermi level. Our calculations show that small band gaps open between the upper flat band and the upper Dirac band at Γ point and between the two Dirac bands at K point, as shown in Fig. 4a–c, which are about 0.354 ~ 0.703 and 0.200 meV, respectively. These small band gaps are due to the weak SOC of the *p*_*z*_ atomic orbit of the TPyB ligands and thus are of the same order as those in δ-graphyne^40^, silicene^41^ and SiC_3_^42^. It is noteworthy that the partial charge density decomposed to the bands (labeled I and II in Fig. 4a) is delocalized with a distribution across the whole lattice (see Fig. 4d,e), which differ fundamentally from those of the defect states with localized density existing in semiconductors^43^,^44^. The delocalized charge density of the dispersionless bands is related to the geometrically frustrated feature of the system on which the lattice hopping is frustrated, leading to topological flat bands with nonzero Chern number^45^. The width of the upper flat band is only 0.374 meV. The nontrivial topology of the Cu-TPyB lattice can be evidenced by the Chern number (*C*) of the spin polarized bands calculated using the Kubo formula:


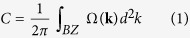


with


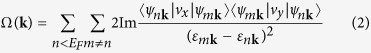


The band I has a nonzero Chern number (*C *= –1), whereas the band II has a zero Chern number (*C *= 0), indicating that the Cu-TPyB lattice is topologically nontrivial. Using the same method, we had also succeed in confirming the existence of a QAH state in a well-designed graphene nanomesh^21^.

We also propose a single-orbit tight-binding (TB) model to reveal the topological aspect of the four bands in the vicinity of the Fermi level which are contributed mainly by the *p*_*z*_ atomic orbits of the light elements in the TPyB component. In this case, the TB Hamiltonian reads as:


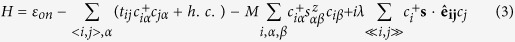


where, 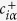 and 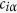 are creation and annihilation operators, respectively, for an electron with spin α on site *i*. *ε*_*on*_ represents the on-site energy for both spin-up and -down channels. *t*_*ij*_ is distance-dependent hopping integral. The last two terms represent the contributions of exchange field and next-nearest intrinsic SOC, respectively. More details can be seen in the SI online. Using appropriate parameters, the TB Hamiltonian reproduces well the four DFT bands, especially the SOC gaps at the Γ and K points (see Fig. 4a–c). This confirms that the opened band gaps in the Cu-TPyB lattice are due to the intrinsic SOC of *p*_*z*_ orbits of the light elements. The distributions of Berry curvatures calculated from the above TB Hamiltonian for the bands I and II in the momentum space are plotted in Fig. 4f–g, respectively. It is obvious that the Berry curvature of the flat band resides mainly around the Γ points, while that of band II is around Γ and K points with opposite signs. We also checked the spin texture of the Cu-TPyB lattice and convinced that the spin direction is normal to the lattice plane, which further confirm that the Cu-TPyB is a magnetic topological insulator. Although the bulk band gaps at the Γ and K points are smaller than the thermal motion energy at room temperature (~26 meV) due to the weak SOC of *p*_*z*_ orbits, they are detectable in experiments at the temperature lower than 8 K, and thus implementable to achieve QAH effect. We noticed that the electron-phonon coupling (EPC) may renormalize the band gaps of some semiconducting materials, such as diamond and trans-polyacetylene^46^,^47^. However, the EPC in the Cu-TPyB is unclear at present. It is uncertain if the small SOC gap of the Cu-TPyB remains implementable in QAH effect as EPC being taken into account.

## Discussion

For the self-assembled structure, mechanical deformations induced by various substrates do exist. It is necessary to discuss the possible effects of electronic coupling to the surface on the electronic properties of Cu-TPyB, e.g. half-metallicity and topological property. We placed the Cu-TPyB lattice on top of hexagonal BN substrate, which is expected to have a weak van der Waals interfacial interaction and right hexagonal symmetry (see Supplementary Fig. S4 online). Our first-principles calculations show that the four distinctive bands of the Cu-TPyB near the Fermi level are well separated from the bands of BN substrate and the main features of half-metallicity and QAH states remain intact. There are still SOC gaps opened between the upper flat band and the upper Dirac band at Γ point and between the two Dirac bands at K point. These results demonstrate the feasibility of attaining the QAH states of the Cu-TPyB on an insulating substrate. We also verified the robustness of the ferromagnetism, half-metallicity and topological nontriviality of the Cu-TPyB at the external strain from -10% to 5%. These exotic properties are well preserved at this stain range. More interestingly, the compress strain can improve the SOC strength of the Cu-TPyB. For example, at the compress strain of –5%, the topologically nontrivial band gaps opened due to SOC increase to 1.43 meV (Γ point) and 0.37 meV (K point), respectively. More details can be seen in the SI online. It is noteworthy that the growth of Cu-TPyB lattice was only achieved on Au(111) substrates in recent experiments^29^. The abundant electronic states of the metal substrates may destroy the half-metal properties of the Cu-TPyB. Transferring the Cu-TPyB to or growing Cu-TPyB on an isolated insulating substrate is necessary for the utilization of Cu-TPyB in spintronic devices.

To conclude, using first-principles calculations combined with a TB model, we theoretically demonstrate that the already-synthesized 2D Cu-TPyB is a half-metal with metallic nature in one spin channel and a large band gap in the other. Two Dirac bands sandwiched by two topological flat bands arise mainly from the *p*_*z*_ atomic orbits of the light elements in the organic ligands and play a decisive role in the exotic properties of the Cu-TPyB. SOC opens small band gaps between the upper flat band and upper Dirac band at the Γ point and between the two Dirac bands at the K point that are experimentally detectable at the temperatures lower than 8 K. The bulk band gaps are topologically nontrivial as confirmed by Berry curvature and Chern numbers. Both the half-metallicity and topological nontrivialty are robust against external strain. These features are quite promising for achieving quantum anomalous Hall effects and device applications.

## Methods

Our first-principles calculations are performed using the plane wave basis Vienna ab initio simulation pack (VASP)^48^,^49^. For the electron-electron interaction, we employ the generalized gradient approximation of Perdew-Burke-Ernzerhof (PBE) functional^50^, which has been widely used to deal with metal-organic systems^19^,^27^. The electronic properties obtained from the PBE functional were also confirmed using a more accurate hybrid Heyd-Scuseria-Ernzerhof (HSE) functional^36^,^37^,^38^. The electron-ion interaction is described by projector-augmented-wave (PAW) potentials. One electron for hydrogen (1s^1^), four electrons for carbon (2s^2^2p^2^), five electrons for nitrogen (2s^2^2p^3^), and eleven electrons for copper (3d^10^4s^1^) are treated as valence electrons. The vacuum region is about 15 Å to avoid mirror interaction between neighboring images. Structural optimizations are performed using a conjugate gradient (CG) method until the remanent force on each atom was less than 0.01 eV/Å. A plane-waves energy cutoff of 500 eV is used on a 1 × 1 × 1 Monkhorst-Pack sampling for CG calculations and on 3 × 3 × 1 for total energy calculations. Electronic spin-polarization is involved in all calculations. Our results are obtained from the above calculation method, except where noted.

## Additional Information

**How to cite this article**: Zhang, X. and Zhao, M. Robust half-metallicity and topological aspects in two-dimensional Cu-TPyB. *Sci. Rep.* **5**, 14098; doi: 10.1038/srep14098 (2015).

## Supplementary Material

Supplementary Information
