# Supplementary material for: Robust half-metallicity and topological aspects in two-dimensional Cu-TPyB
Source: Sci Rep. 2015 Sep 14;5:14098. doi: 10.1038/srep14098 (PMC4568493; doi:10.1038/srep14098)
Supplement: Supplementary Information [file srep14098-s1.pdf]

Supporting Information for

Robust half-metallicity and topological aspects in two-dimensional Cu-TPyB

Xiaoming Zhang and Mingwen Zhao*

School of Physics and State Key Laboratory of Crystal Materials, Shandong University, Jinan, Shandong, 250100, China

E-mail: zmw@sdu.edu.cn Phone: 86-531-88364655

This Supporting Information (SI) is meant to support some of the explanations made in the main text. In the first two parts, detailed descriptions of Monte Carlo (MC) simulations and a tight-binding (TB) approximation are presented. The robustness of half-metallicity and topological insulator states of Cu-TPyB on a BN substrate or under mechanical deformations were discussed in the last part.

Part I. Monte Carlo (MC) simulations.

As mentioned in the main text, the stability of the FM ordering in Cu-TPyB was evaluated by using Monte Carlo (MC) simulations within the *Ising* model: $H = -J_0 \sum_{\langle i,j \rangle} \hat{m}_i \times \hat{m}_j$, where $\hat{m}_i$ and $\hat{m}_j$ are the local magnetic moments at the nearest-neighbor sites *i* and *j*. The nearest-neighbor exchange parameter, J_0, of local magnetic moments can be determined from the formula of $J_0 = \Delta E / (6 \times m_i \times m_j)$, where ΔE is the energy difference between the FM and AFM states, $\Delta E = E_{FM} - E_{AFM}$, obtained from first-principles calculations. Considering the magnetic moment of 3.0 (or 0.0) μ_B distributing on two local magnetic components that are FM (or AFM) coupled with each other, we estimated a scope of J_0 when $\hat{m}_i$ ranging from 1.5 μ_B to 1.0 μ_B, which is 4.40~4.95 meV. We employed a 50×50 supercell containing 5000 local magnetic moments. The simulations lasted for 1×10^9 loops, in which each spin is changed accordingly. The temperature-dependent magnetic moments per unit cell are then obtained from MC simulations as shown in Figure S1. The magnetic moment equals 0.0 μ_B when the temperature exceeds 150 K. To further confirm the Curie temperature (T_c), we calculated the heat capacity (C_v) of the system using the following expression: $C_v = \lim_{\Delta T \to 0} \frac{\Delta E_T}{\Delta T}$, where ΔE_T is the change of the total energy of the system as the temperature increases from T to $T + \Delta T$. The calculated C_v as a function of temperature is shown in the Figure S2, which indicate that the Curie temperature (T_c) is in the range of 137~151 K, and that the ferromagnetic-paramagnetic transition is a second order phase transition. Although this MC simulation only gives a rough estimation of the Curie temperature, it is consistent with the high stability of the FM state at the temperatures lower than 151 K, which is at the same temperature range as that predicted in the Mn–Pc sheet[1].

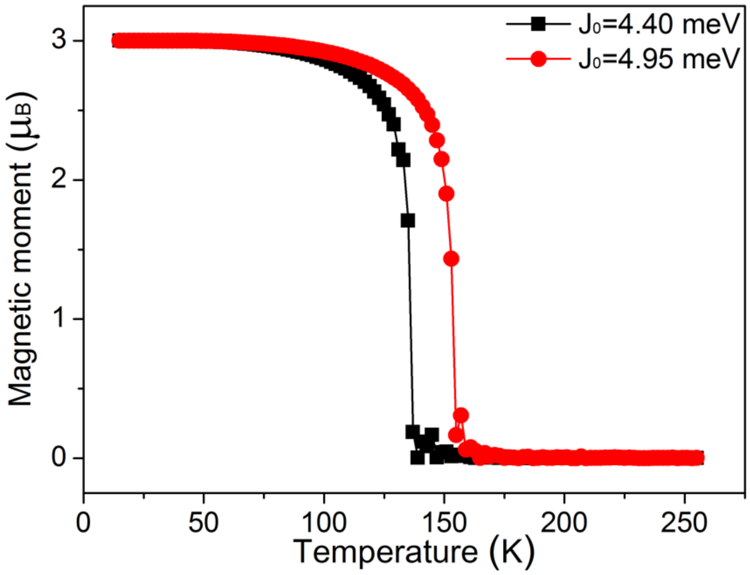

Figure S1. Temperature-dependent magnetic moment per unit cell obtained from the MC simulations with different nearest-neighbor exchange parameter.

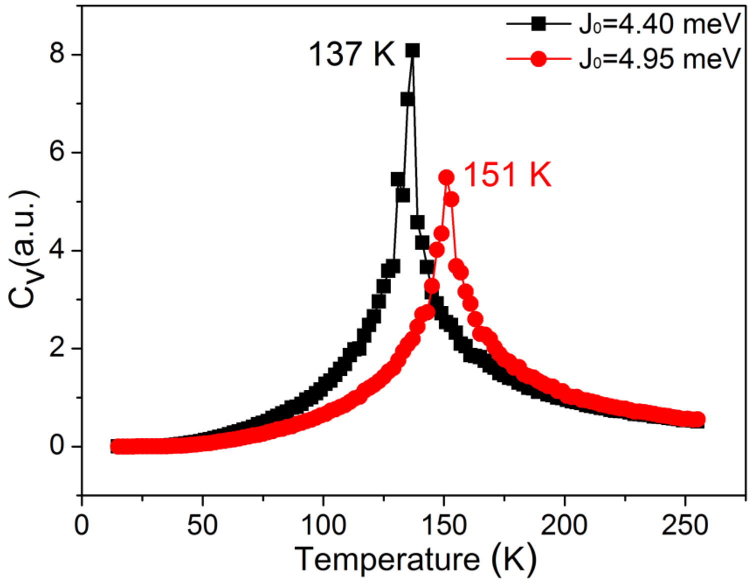

Figure S2. Variations of heat capacities as a function of temperature obtained from the MC simulations based on the *Ising* model.

Part II. Tight-binding (TB) approximation.

The single-orbital (p_z) TB Hamiltonian of the Cu-TPyB lattice reads as

$$H = \varepsilon_{on} - \sum_{<i,j>,\alpha} (t_{ij} c_{i\alpha}^{+} c_{j\alpha} + h.c.) - M \sum_{i,\alpha,\beta} c_{i\alpha}^{+} s_{\alpha\beta}^{z} c_{i\beta} + i\lambda \sum_{<<i,j>>} c_{i}^{+} \mathbf{s} \cdot \hat{\mathbf{e}}_{\mathbf{ij}} c_{j}$$

where, $c_{i\alpha}^{+}$ and $c_{i\alpha}$ are creation and annihilation operators, respectively, for an electron with spin α on site *i*. ε_{on} is onsite energy for both spin up and down channels. t_{ij} is distance-dependent hopping integral. *M* and *λ* are the strength of the exchange field and the next-nearest intrinsic SOC of p_z atomic orbitals, respectively. **s** are the spin Pauli matrix and the unit vector $\hat{\mathbf{e}}_{ij}$ is defined as: $\hat{\mathbf{e}}_{\mathbf{ij}} = \frac{\mathbf{d}_{\mathbf{ij}}^{\mathbf{1}} \times \mathbf{d}_{\mathbf{ij}}^{\mathbf{2}}}{\left|\mathbf{d}_{\mathbf{ij}}^{\mathbf{1}} \times \mathbf{d}_{\mathbf{ij}}^{\mathbf{2}}\right|}$, here $\mathbf{d}_{\mathbf{ij}}^{\mathbf{1}}$ and $\mathbf{d}_{\mathbf{ij}}^{\mathbf{2}}$ are the bond vectors along the two bonds that the electron traverses when going from atom *j* to *i*. Taking the influences of Copper atoms on the electron transition into account, the hopping integrals t_{ij} are classified into two categories. One is the electronic hopping between two nitrogen atoms connected by Cu, defined as $t_{ij} = t_n$. The other is the distance-dependent hopping between the light atoms given as the following form: $t_{ij} = t_0 \exp(q(1 - d_{ij}/d_0))$, here t_0 is nearest-neighbor (NN) hopping integral between the NN atoms with the bond length of d_0. d_{ij} is the distance between *i*-th and *j*-th atoms. The above parameters are partly marked in Figure S3.

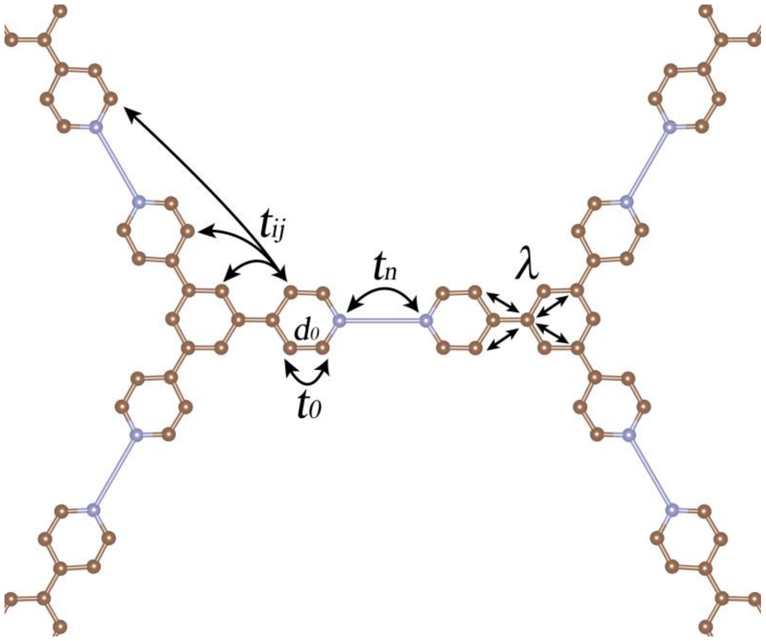

Figure S3. The parameters used in the tight-binding model.

The TB band lines reproduce well the four bands obtained from the DFT calculations in the vicinity of the Fermi level with the optimal parameters of

$\varepsilon_{on} = -2.875$ eV, $t_n = -0.980$ eV, $t_0 = -3.000$ eV, $q = 1.240$, $d_0 = 1.41$ Å, and $M = -0.171$, respectively, as shown in Figure 4a. When the SOC strengths are set to λ=0.00014 and 0.00057 respectively for K and Γ points, band gaps are opened up, as shown in Figure 4b and 4c in the main text.

Part III. Robustness of half-metallicity and topological insulator states of Cu-TPyB lattice on a BN substrate or under mechanical deformations.

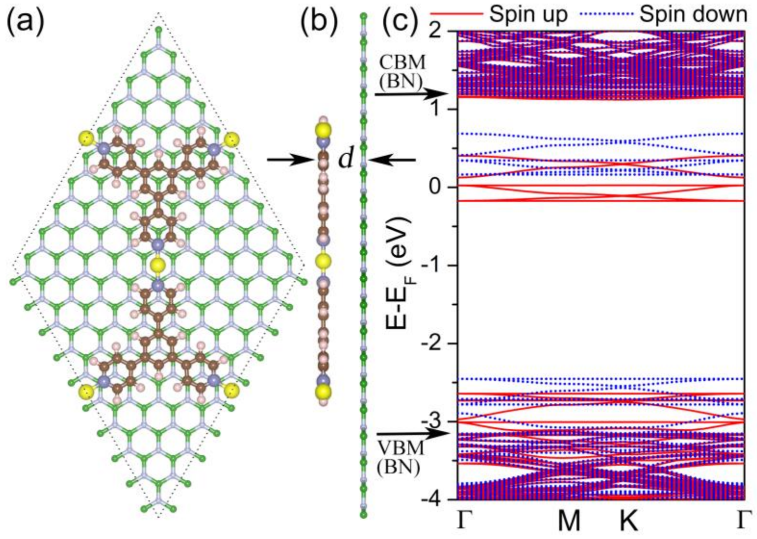

Figure S4. (a) Top view, (b) side view and (c) spin-resolved band structures of the Cu-TPyB lattice on BN substrate.

Considering the Cu-TPyB is formed on a supporting surface in experiments, it is crucial to discuss the possible effects of electronic coupling to the surface on the electronic properties of the Cu-TPyB. Due to the metal substrates with the abundant states near the Fermi level that might conceal the four distinctive bands of Cu-TPyB, we placed the lattice on top of hexagonal BN substrate, which is expected to have a weak van der Waals interfacial interaction and right hexagonal symmetry, as shown in Figure S4a and S4b. After structural relaxation, the interlayer spacing is d=3.51 Å, indicating the interlayer interaction belongs to van der Waals interaction. The electronic band structure of the Cu-TPyB/BN heterostructure is plotted in Figure S4c.

It is clear that the four distinctive bands of the Cu-TPyB in the vicinity of the Fermi level are well separated from the CBM and VBM of the BN substrate, and resemble those of the standalone Cu-TPyB very well. The main features of half-metallicity and QAH states remain intact. There are still SOC gaps opened between the upper flat band and the upper Dirac band at Γ point and between the two Dirac bands at K point. These results demonstrate the feasibility of attaining the QAH states of the Cu-TPyB on a substrate.

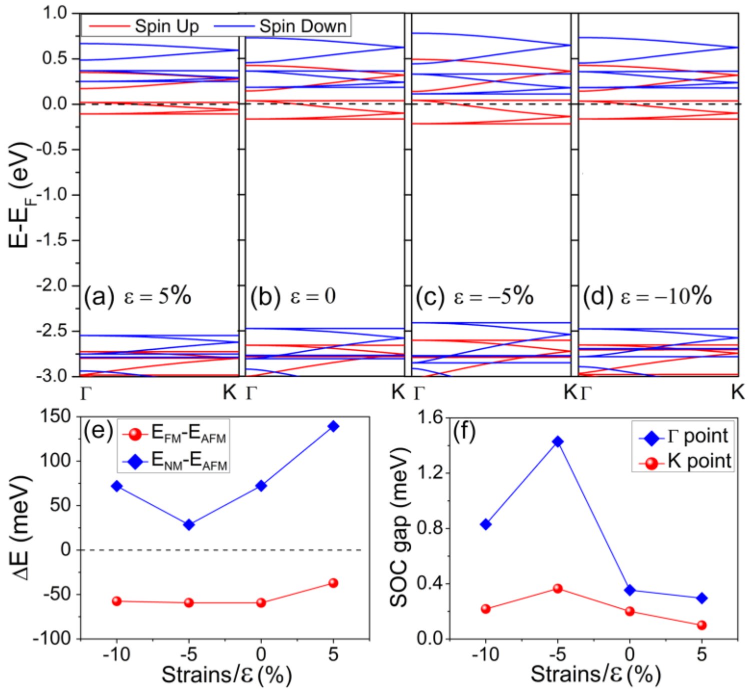

Figure S5. (a,b,c,d) Spin-polarized band structures of Cu-TPyB under different strains. (e) The energy differences between different magnetic configurations and (f) the band gaps opened by SOC at the Γ or K points near the Fermi level fluctuate with the change of the tensile strain.

Because the mechanical deformation is inevitable in device application of Cu-TPyB lattice, we investigate the modification of geometric and electronic structures of the Cu-TPyB in response to in-plane biaxial strain. The biaxial tensile strain is defined as $\varepsilon = (a\text{-}a_0)/a_0$, where a and a_0 are the lattice constants of the strained and equilibrium Cu-TPyB framework. The energy differences between FM and AFM states, and between NM and AFM states fluctuating with the change of the strain are shown in Figure S5e. We can clearly see the FM configuration is still lower in energy than the

AFM one, and the NM states always possess highest energies. The calculated band structures (see Figure S5a, S5b, S5c, S5d) indicate the half-metalicity is well preserved in the strained Cu-TPyB, and the width of the Dirac bands near the Fermi level narrows down with the tensile strain increasing from -5% to 5%. The topological nontriviality is also preserved in these strained structures. The band gaps opened by SOC at the Γ and K points near the Fermi level are shown in Figure S5f. Except for the tensile strain of −10%, we can easily conclude that the shortening of the lattice constant will improve the strength of SOC and enhance the interaction between Cu and TPyB ligands.

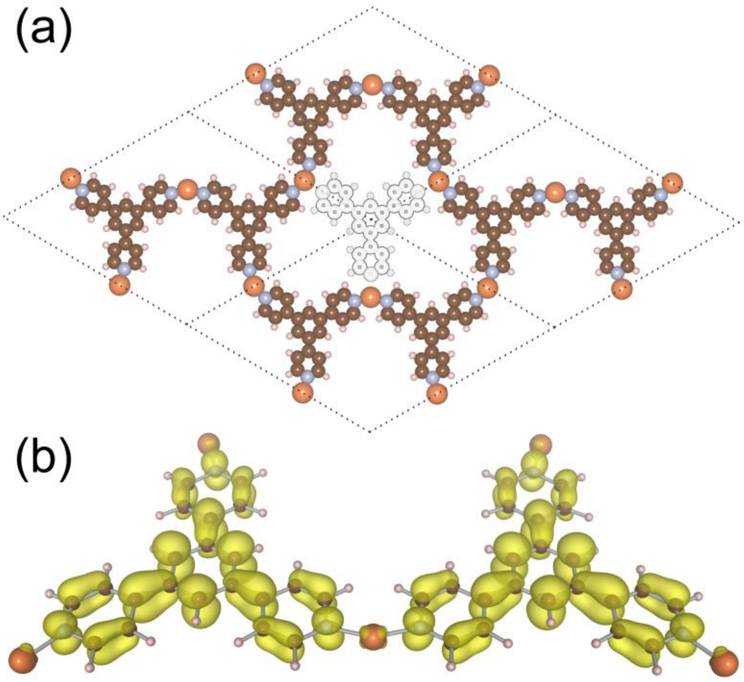

Figure S6. (a) Schematic representation of Cu-TPyB and (b) the partial charge density isosurfaces decomposed to the two flat bands and the two Dirac bands in the energy range from E_F − 0.2 to E_F + 0.1 eV under the strain of ε = −10%. The inset in the center of (a) is a hypothetical TPyB ligands to indicate the three-fold Cu-pyridyl coordination. The isosurface value in (b) was set to 0.002 $Å^{-3}$.

To explore the reasons of the abnormal phenomenon at tensile strain of −10%, we checked the structure of Cu-TPyB and found it undergoes an in-plane reconstruction, leading to a low symmetry configuration, as shown in Figure S6a. This is consistent with the prediction that Cu atom can bond to three TPyB ligands and Cu-pyridyl can be altered to a three-fold coordination under high compression[2]. The reconstruction

leads to a slight shift in the relative positioning of the partial charge density decomposed to the four bands in the vicinity of the Fermi level, as shown in Figure S6b. The corresponding band structure (see Figure S5d) shows the compressed framework remains a half-metal that still stem from the p_z atomic orbitals of light elements in the TPyB section. The in-plane reconstruction does not fundamentally affect the properties of Cu-TPyB.

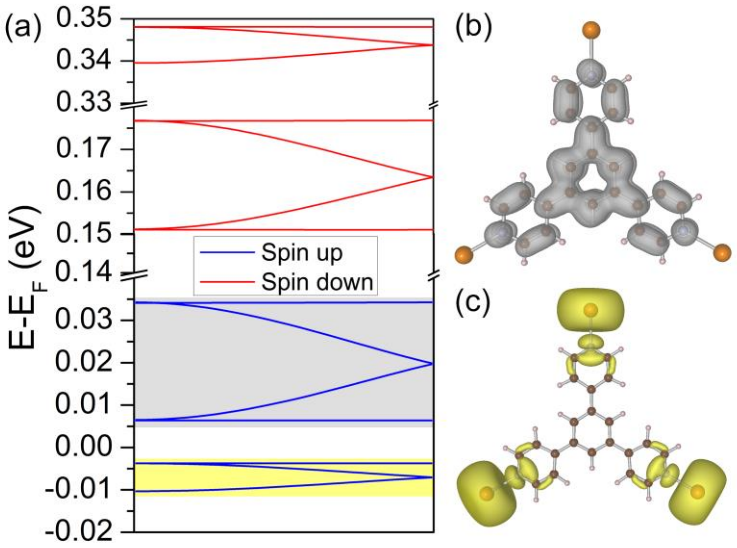

Figure S7. (a) Spin-polarized band structure of Cu-TPyB at the tensile strain of ε = 10%. (b, c) The partial charge density isosurfaces decomposed (b) to the four bands at the grey region (c) and to the three bands at the yellow region in (a). The isosurface value was set to 0.002 $Å^{-3}$.

With the tensile strain further increasing, e. g. ε = 10%, the Cu-TPyB becomes a spin-polarized semiconductor with a trivial band gap of 7.42 meV, as shown in Figure S7a. The magnetic moment per unit cell is still 3.0 μ_B, but fundamentally different from the previous discussions. The partial charge density decomposed to the three spin-up bands at the yellow region in Figure S7a is plotted in Figure S7c, which shows the magnetic moment comes mainly from the copper atoms when considering the corresponding three unoccupied spin-down bands. Total energy comparison indicates that the FM ordering is still more stable than the AFM and NM states by about 136.98 and 173.72 meV/unit cell, respectively. There is no electrons occupied the four bands at the grey region, the p_z-orbits of carbon and nitrogen atoms (see

Figure S7b), indicating the larger tensile strain preventing the electrons of Cu from transferring to the TPyB ligands. This phase transformation can be attributed to the large lattice constant weakening the interaction between Cu and TPyB ligands.

1. Zhou, J.; Sun, Q., Magnetism of phthalocyanine-based organometallic single porous sheet. *J Am Chem Soc* 2011, 133, 15113-9.

2. Liu, J.; Lin, T.; Shi, Z.; Xia, F.; Dong, L.; Liu, P. N.; Lin, N., Structural transformation of two-dimensional metal-organic coordination networks driven by intrinsic in-plane compression. *J Am Chem Soc* 2011, 133, 18760-6.
